# Improvements to the Sink Strength Theory Used in Multi-Scale Rate Equation Simulations of Defects in Solids

**DOI:** 10.3390/ma13112621

**Published:** 2020-06-09

**Authors:** Tommy Ahlgren, Kalle Heinola

**Affiliations:** 1 Department of Physics, University of Helsinki, P.O. Box 43, FI-00014 Helsinki, Finland; 2 International Atomic Energy Agency, P.O. Box 100, 1400 Vienna, Austria; k.heinola@iaea.org

**Keywords:** rate equations, sink strength, rate theory, defect dynamics, trapping, TDS, multi-scale modeling, 02.30.Jr, 05., 05.10.-a, 05.10.Ln

## Abstract

The application of mean-field rate theory equations have proven to be a versatile method in simulating defect dynamics and temporal changes in the microstructure of materials. The reliability and usefulness of the method, however, depends critically on the defect interaction parameters used. In this study, we show that the main interaction parameter, the sink strength, intrinsically depends on the detrapping, or the dissociation process itself. We present a theory on how to determine the appropriate sink strengths. The correct sink strength required for a detrapping defect, is considerably larger than the values commonly used, and thus should not be neglected.

## 1. Introduction

The physical and mechanical properties of materials are largely determined by their microstructure, and defect and impurity concentrations. To understand and control these changes during aging, ion irradiation or annealing, a simulation technique extending to long time and length scales is required. Among simulation techniques able to fulfil these challenging demands are the mean-field rate theory equations (RE) and the kinetic Monte Carlo (KMC) methods. KMC is a stochastic simulation method, where all the dynamic properties and reactions for all incorporating defects have to be known a priori. A single time step used in a KMC simulation, however, might be of the order of 10^−12^ s for fast occurring processes [1], which will restrict the accessible time and the amount of defect concentrations studied with this method.

In the mean-field REs, all the relevant defect mobilities and reactions are collected to a set of non-linear partial differential equations that are temporally and spatially solved [2,3,4,5]. The RE methodology has been extensively used for simulating various dynamic processes in solid materials. These studies include trapping simulations of helium (He) and hydrogen (H) isotopes in vacancies [6,7], clustering of irradiation-induced vacancies and self-interstitials [3,8,9,10], He and H bubble formation [11,12], H diffusion and retention [13], precipitation, swelling, and segregation [14,15,16], fusion edge-localized plasma modes simulations [17], H isotope exchange [18,19,20], irradiation-induced amorphization [21], and simulation of thermal desorption spectrometry (TDS) profiles [22,23,24,25] to mention just a few.

The parameters for detrapping or dissociation processes can be determined by reaction rate theory [26] and transition state theory [27,28]. The trapping or annihilation processes are described by the so-called sink strength parameter [2,3,15]. Sink strengths have been determined analytically for various symmetric defects or traps including spherical traps, dislocation lines, and grain boundaries [3,14,29,30]. Finding the sink strength for arbitrarily shaped traps the conventional Monte Carlo (MC) method can be used [31,32,33].

The main advantage of the RE is that it is a very fast method for simulating processes taking place for extended time and length scales. The usefulness of the simulation results, however, depends critically on the defect interaction parameters, i.e., there is a critical requirement that the sink strengths applied, describe the physical processes correctly. In this paper, we show that the sink strength depends decisively on the detrapping or the dissociation process. We further derive the equations highlighting the requirements how the sink strengths and REs should be modified accordingly. Finally, we make test simulations to compare the results obtained using different sink strength theories.

## 2. Results

### 2.1. Theory

The presentation of the rate equation and sink strength theories are given in Refs. [2,3,14,15]. The governing idea is to relate the time evolution of any defect concentration to the trapping and detrapping processes. A simplified rate equation with only one kind of trap, excluding boundary diffusion and source terms for brevity, is given by

(1) $$\begin{array}{cc} \frac{dc}{dt} & =-c_{e}\oint F^{in}dA+c_{f}\oint F^{out}dA\\ & =-Dkc+E, \end{array}$$

where $c_{e}$ and $c_{f}$ are the concentrations of the empty and filled traps, $F^{in}$ and $F^{out}$ are the trapping and detrapping fluxes of defect *c* to and from a trap, respectively. Parameters $c_{e}\oint F^{in}dA$ and $c_{f}\oint F^{out}dA$ denote the trapped and detrapped defect concentrations per second, where *A* is the trap surface area. These integrals are impractical to use and are substituted in the RE method by terms −Dkc and +E, respectively, as presented in Equation (1). *D* is the defect diffusion coefficient, *k* the sink strength, and *E* is the thermal emission (detrapping) of defects *c* from the filled traps $c_{f}$. According to rate theory, *E* can be represented as

(2) $$\begin{array}{c} E=c_{f}\nu \;\exp\left(-\frac{E_{t}}{k_{B}T}\right), \end{array}$$

where ν is the detrapping attempt frequency, $E_{t}$ ($\approx E_{b}+E_{m}$) is the trapping or dissociation energy, $E_{b}$ is the binding energy and $E_{m}$ the defect migration barrier, $k_{B}$ is the Boltzmann constant, and *T* the absolute temperature.

**Figure 1:**
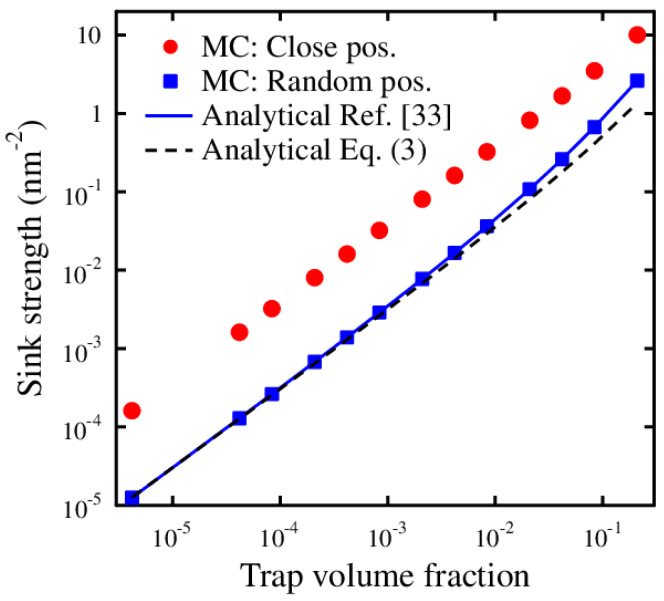
The sink strengths for spherical traps calculated by Monte Carlo (MC) and analytical methods. The position for the defect in the simulation cell is either random or at a close distance (0.05 nm) from a trap boundary. The sink strength for defects with close to trap initial position is greatly enhanced compared to the ones with the random initial position.

Usually, the sink strengths, *k*, are determined analytically or by applying the MC method. The detrapping attempt frequency and the trapping energy are obtained with atomistic simulations or with experiments. As an example of analytical sink strength, and to be used later in this work, the widely applied sink strength for spherical traps is given by Brailsford and Bullough [29]

(3) $$\begin{array}{c} k=4\pi R_{t}c_{e}\left(1+R_{t}\sqrt{k}\right), \end{array}$$

where $R_{t}$ is the trapping radius and $c_{e}$ the trap concentration. Most of the sink strengths used in the literature are defined for randomly distributed defects in the material which result, for instance, from ion irradiation of the material. The same sink strengths are usually used irrespective of how the defects enter the material. We will, however, now show that the sink strengths influencing detrapped defects, are greatly enhanced compared to sink strengths of defects entering the material through irradiation. This is done by comparing sink strengths for defects having the initial position either randomly distributed in the cell, or in close proximity of a trap boundary. The fast MC method [33] was used in calculating the sink strengths *k* for different defect positions with respect to the trap. The results are shown in Figure 1 as a function of the relative amount of traps, i.e., the trap volume fraction.

In the MC calculations, the traps simulated are spherical with a trapping radius of $R_{t}$ = 1.0 nm and the trap concentration is varied between 6 × 10^−6^ − 0.4 nm^−3^. The defect jump length is set to 0.1 nm. The number of defects for each trap concentration is 10 × 10^6^ giving a statistical error of about 0.03% for the sink strengths [33]. The results show that defects with an initial position close to a trap have a large probability to be trapped in the adjacent trap. Thus, their sink strengths are much larger than the sink strengths for defects with the random position further away from any trap. In addition, in Figure 1 are shown the analytical sink strength values as obtained from Ref. [33] and compared against the values obtained using Equation (3). The analytical results show minor deviation features only at higher trap volume fractions, which will be discussed later in this work.

**Figure 2:**
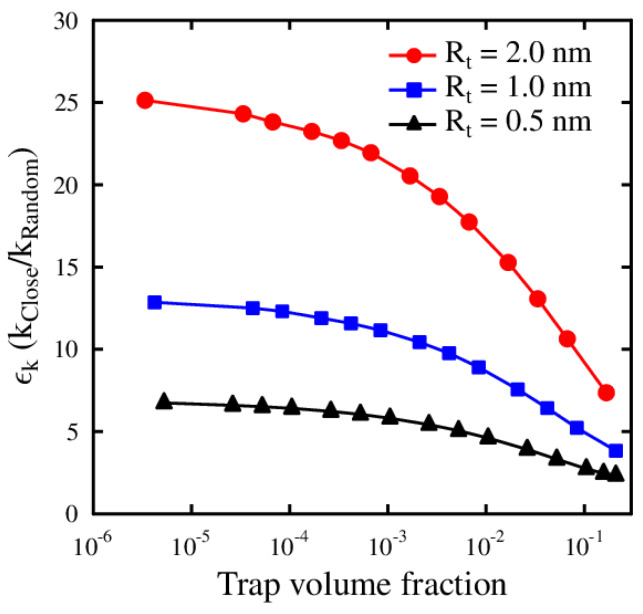
The enhancement factor $\epsilon_{k}$ for sink strengths as a function of trap volume fraction for three different trapping radii. At high trap concentrations, the probability of a defect to be trapped to other than the nearest trap increases, which is seen as a decrease in the $\epsilon_{k}$ values.

To formulate the aforementioned position-sensitive effect of sink strengths, we present the modified sink strength, which incorporates the position of the defect with respect to the trap. We introduce a dimensionless enhancement factor $\epsilon_{k}$ as $\epsilon_{k}$ = $k_{close}/k_{rand}$, which is defined as the sink strength for defects located close to the trap divided by the sink strength for defects with random initial position. Figure 2 shows this enhancement factor as a function of trap volume fraction for three different trapping radii: 0.5, 1.0, and 2.0 nm. The results for trapping radius 1.0 nm are calculated from the MC sink strength results presented in Figure 1.

It can be seen that the enhancement factor increases with the trap size, which reflects the fact that the trapping probability for the defect close to the trap increases as the trap size increases. The enhancement factor approaches a maximum value as the trap volume fraction approaches zero. As the trap volume fraction increases, the trap concentration becomes higher, and the probability for the defect to be trapped to another trap than the nearest one increases, leading to a smaller enhancement factor. For very high trap volume fractions the $\epsilon_{k}$ approaches unity, when the sink strengths $k_{close}$ and $k_{rand}$ approach equal values. The key point is that the sink strengths for defects vary depending on their lattice position. These positions may be randomly distributed as a result of irradiation, or close to an empty trap due to detrapping. With this in mind, we revisit the RE theory as presented by Equation (1). At equilibrium (dc/dt=0) the trapping and detrapping rates are in balance and Equation (1) yields

(4) $$\begin{array}{c} Dkc=E=c_{e}\oint F^{in}dA=\epsilon_{k}c_{f}\oint F^{out}dA, \end{array}$$

which takes into account the sink strength enhancement $\epsilon_{k}$. From this we get

(5) $$c_{f}\oint F^{out}dA=E/\epsilon_{k},$$

which together with the sink strength from Equation (1) yields

(6) $$\begin{array}{cc} k & =\frac{1}{D\;c}\left[c_{e}\oint F^{in}dA-E/\epsilon_{k}+E\right]\\ & =\frac{c_{e}}{D\;c}\oint F^{in}dA+\frac{E(1-1/\epsilon_{k})}{D\;c}\\ & =k_{rand}+\frac{E(1-1/\epsilon_{k})}{D\;c}. \end{array}$$

The first term in Equation (6), $k_{rand}$, is the usual random position sink strength [3,15]. The second term contains the factor $\epsilon_{k}$, which enhances the sink strength when detrapping is activated. With no detrapping, or if the enhancement factor is one, the sink strength reduces to the one conventionally used. The full rate equation, including diffusion and the source of defects (S) originating from the irradiation process, now reads

(7) $$\begin{array}{ccc} \frac{dc}{dt} & = & D\nabla^{2}c-Dkc+E+S, \end{array}$$

where the *k* and *E* are given as in Equations (2) and (6), respectively. An alternative formulation that gives the right equilibrium condition is obtained when Equation (6) is inserted in Equation (7)

(8) $$\begin{array}{ccc} \frac{dc}{dt} & = & D\nabla^{2}c-Dk_{rand}c+E/\epsilon_{k}+S. \end{array}$$

### 2.2. Rate Equation Simulations

To highlight the effect of using different sink strength theories, we perform the following RE simulation for detrapping, trapping, and diffusion processes during annealing. The simulation conditions have been chosen such, that the corresponding KMC simulation is possible to be carried out allowing comparison. In these simulations, we have a 200 nm thick material layer with a Gaussian-shaped trap profile located at a mean depth of 50 nm from the surface and with a standard deviation (SD) 10 nm. The maximum trap concentration value at the mean depth is set to 1 × 10^18^ cm^−3^. Initially, the traps are filled with one defect per trap, and the trapping radius *R_t_* is set to 1.0 nm. The defect detrapping attempt frequency ν is 5 × 10^12^ Hz and the binding energy *E_b_* = 0.8 eV, as used in Equation (2). The temperature at the beginning of the simulation is set to 300 K, and it increases linearly with a rate of 50 K/s up to 800 K during the 10 s simulation. As the temperature is rising in the simulation, detrapping, and diffusion processes of defects start to take place. Some defects are retrapped, but some reach the surface and leave the simulation layer. This flux of defects to the surface is monitored and the corresponding results obtained with RE and KMC methods are compared. This simulation represents the popular TDS method frequently used in the experiments and the corresponding computational analyses. Processes taking place on the surface have been omitted in the present simulations. Thus, the defect flux to surface is taken directly as the flux of defects crossing the simulation layer boundary. The defect diffusion parameters used are: jump length is 0.1 nm, jump frequency is 5 × 10^12^ Hz, and the migration barrier $E_{m}$ = 0.25 eV. The detrapping distance for the defects from the traps is chosen as 0.05 nm. In this simulation, the RE is represented in 1D and reads

(9) $$\begin{array}{ccc} \frac{dc}{dt} & = & D\frac{d^{2}c}{dz^{2}}-Dkc+E, \end{array}$$

with *z* being the depth coordinate. The different sink strengths *k* and detrapping terms *E* used are given in Table 1. The difference in using Equation (3) or the sink strengths from Ref. [33] for $k_{rand}$ in the table is very small, as seen in Figure 1, due to the low trap volume fraction used in the simulation.

**Figure 3:**
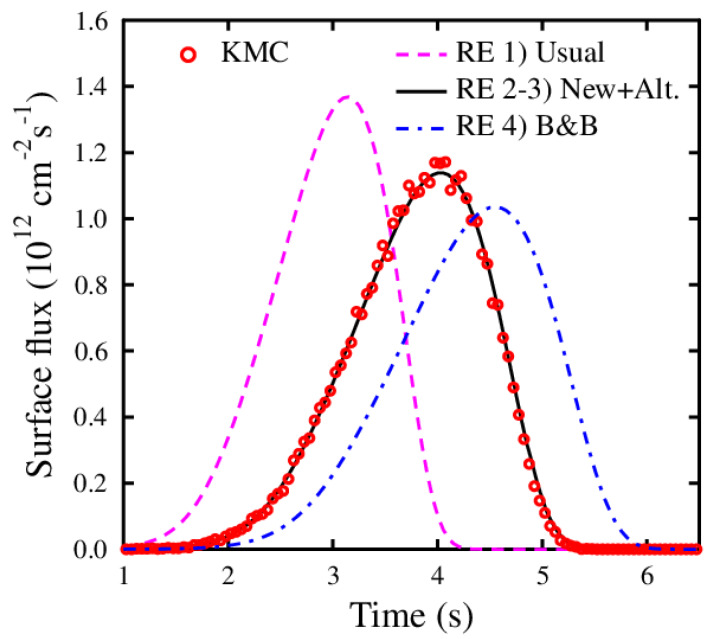
The front surface defect flux comparison between the kinetic Monte Carlo (KMC) and rate theory equations (RE) simulations using different sink strength theories (see text and Table 1).

Figure 3 illustrates the resulted front surface fluxes of defects as obtained with the RE and KMC simulations. It is apparent from the results that the simulated TDS peak (i.e., the calculated defect flux through the front surface) occurs too fast with the usual RE formalism. In view of this study, this is to be expected, because the usually used random sink strength does not take into account the enhanced retrapping due to the defects being detrapped close to the empty traps. The results obtained with the new theory Equation (7) and the alternative formulation Equation (8) agree nicely with the KMC simulation. Moreover, the widely used formulation for sink strength and detrapping, (Table 1) [29], results in a delayed TDS release peak.

Equation (7) with the actual sink strength (given in Equation (6)) should be used if the correct trapped and detrapped defect rates (defect/s) are wanted from the simulation. In this case, the KMC and RE give the same results for the individual trapped and detrapped number of defects per second. Looking at the change in the defect concentration (*c*) as a function of time, however, both Equations (7) and (8) give the same result. The term ($E/\epsilon_{k}$) in Equation (8) is actually the detrapped defects minus the retrapped defects due to the enhanced trapping. The reason for using Equation (8), is that it is simpler and that the time steps in the simulation can be chosen bigger.

This example underlines the importance of the presented theory, i.e., not using enhanced sink strengths, which take into account the defect lattice position, can lead to totally different results. In many studies, the different trapping parameters are fitted so that the RE simulation agrees with the experiment. With the wrong sink strength theory, these fitted parameters are questionable and may have no scientific value.

**Figure 4:**
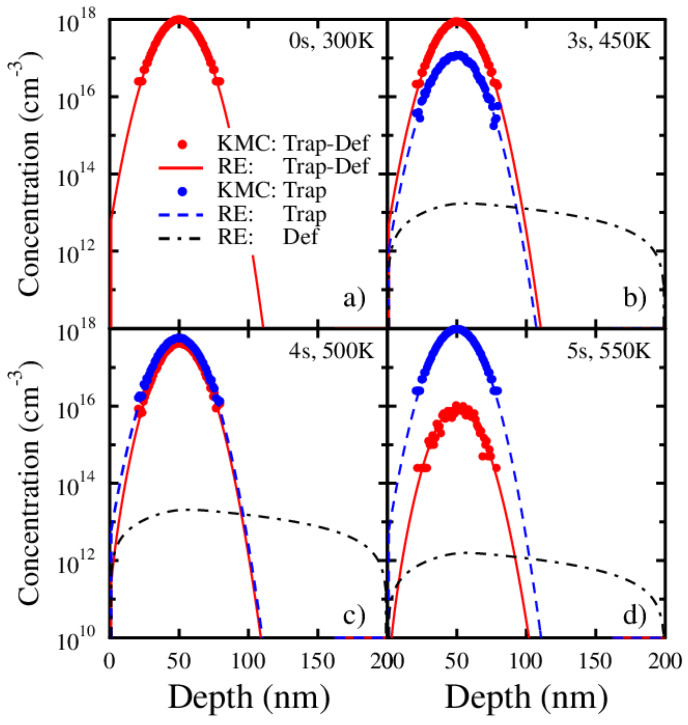
The depth profiles for the KMC and RE simulations at four different times and temperatures during the TDS simulation. At the beginning, at time 0 s, (**a**) the temperature is 300 K, and there is only the filled trap profile (Trap-Def). The temperature increases linearly with time (50 K/s) and at 3 s, (**b**) some defects (Def) have detrapped, leaving empty traps (Trap). At 4 s, (**c**) there are more empty than filled traps, and at 5 s, (**d**) only a few filled traps are left. Note that the number of defects (Def) for the KMC simulation are too few to determine any meaningful defect depth profiles.

Further, in Figure 4 are shown snapshots of the depth profiles of traps and defects at different times during the performed RE and KMC simulations. The chosen times are the beginning, t = 0 s (Figure 4a), at t = 3 s (450 K) where detrapping has started (Figure 4b), at t = 4 s (500 K) where the defect flux through the front surface is at its maximum (Figure 4c), and at t = 5 s (550 K) where almost all traps have been emptied and the defect flux through the front surface approaches zero (Figure 4d).

The profiles are named as follows: traps with defects (Trap-Def), empty traps (Trap), and mobile free defects (Def). From Figure 4 we can see the RE and KMC simulations agreeing perfectly throughout the whole comparable concentration region. The computing time, however, limits the number of traps and defects that can be studied with the KMC method. This can be seen as the lower concentration limit of about 1 × 10^14^ defects/cm^3^ shown in Figure 4d), whereas the corresponding maximum concentration of the diffusing defect profile, obtained with the RE simulation, is well below 1 × 10^14^ cm^−3^. Consequently, it is not possible to deduce the temporal defect depth profiles from the KMC simulation. The perfect agreement of the TDS peaks obtained with the RE and KMC methods (Figure 3), however, indicates that the mobile defect profiles will continue to match at lower concentrations, if the number of defects in the KMC simulation would be sufficient.

It must be mentioned that even though the simulation set-up is chosen favourable for the KMC technique, the RE simulation is about five orders of magnitude faster than the KMC simulation (about 10 s compared to 11 days, respectively). The fact that the number of free defects in the KMC simulation is too small to be used in obtaining any useful defect depth profiles is a serious shortcoming. Often it is very crucial to examine the depth profiles in order to understand the different aspects of the experimental findings, and in obtaining other wanted physical quantities from the simulations.

## 3. Discussion

The results of this study show that the sink strengths depend critically on the position-related detrapping or dissociation processes. Therefore, the numerous simulations including detrapping, done for about 50 years are probably not entirely accurate. The new sink strength formulation needs the new enhancement factor parameter to be determined, which is obtained using, for instance, the MC method. Including the detrapping effect in the sink strength interaction parameters will increase the usefulness of the RE method. The RE simulations should now give more reliable defect and trap parameters, and the long length and time scale microstructure simulations should be more accurate.

## 4. Materials and Methods

The authors declare that the data supporting this study and the MC and RE codes are available from the corresponding author upon request.

## Figures and Tables

**Table 1:** The sink strengths and detrapping terms used for the RE test simulations. $k_{rand}$ is given by Equation (3).

	Sink Strength (*k*)	Detrapping (*E*)
(1) Usual	$k_{rand}$	$c_{f}\nu \;\exp\left(-E_{t}/k_{B}T\right)$
(2) New	$k_{rand}+E\left(1-1/\epsilon_{k}\right)/\left(Dc\right)$	$c_{f}\nu \;\exp\left(-E_{t}/k_{B}T\right)$
(3) New (Alt) ^*a*^	$k_{rand}$	$c_{f}\nu \;\exp\left(-E_{t}/k_{B}T\right)/\epsilon_{k}$
(4) B&B ^*b*^	$k_{rand}$	$4\pi R_{t}c_{f}\left(1+R_{t}\sqrt{k}\right)D\;\exp\left(-E_{b}/k_{B}T\right)$

^*a*^ Alternative formulation Equation (8). ^*b*^ Brailsford and Bullough [29].
